# *Mycobacterium phlei* Vertebral Osteomyelitis

**DOI:** 10.5435/JAAOSGlobal-D-18-00069

**Published:** 2019-12-05

**Authors:** Alan W. McGee, Chase S. Dean, Ashley Ignatiuk, Carla Savelli, Christopher J. Kleck

**Affiliations:** From the Department of Orthopaedic Surgery (Dr. McGee, Dr. Dean, and Dr. Kleck), The University of Colorado, Aurora, CO; the Division Plastics and Reconstructive (Dr. Ignatiuk), Department of Surgery, Rutgers New Jersey Medical School, Newark, NJ; and the Department of Infectious Diseases (Dr. Savelli), The University of Colorado School of Medicine, Aurora, CO.

## Abstract

A report of an instance of vertebral osteomyelitis secondary to an uncommon pathogen, *Mycobacterium phlei*.

**Summary:**

*Mycobacterium phlei* is a rapidly growing nontuberculous osteomyelitis which is typically nonpathogenic with only four reported cases of human infection. Diagnosing infections related to nontuberculous mycobacteria (NTM) is difficult and can often be delayed as conventional microbiologic tests are inadequate. Currently, there are no consensus guidelines concerning the treatment of vertebral osteomyelitis caused by NTM. A 45-year-old man presented with chronic back pain and bilateral lower extremity radicular symptoms status-post lumbar fusion with previous deep infection. CT scan demonstrated incomplete union after fusion. He underwent irrigation and débridement on March 15, 2016, with tissue culture and biopsy. Given negative cultures and completion of a 6-week course of intravenous antibiotics, on May 3, 2016, he went for implant removal and repeat instrumentation. During the same hospitalization, deep spinal fluid acid-fast bacilli culture from March 15, 2016, came back positive at 8 weeks, identified as *Mycobaterium phlei*. He was started on an empiric 4-drug regimen for NTM which he continued for 12 months. There has been no recurrence of infection to date.

**Discussion:**

This case serves as the first description of *M. phlei* osteomyelitis of the spine and as a reminder that proper diagnosis of infectious etiologies is necessary for adequate treatment.

A 45-year-old man presented with chief complaints of chronic back pain and bilateral lower extremity radicular symptoms in November 2015. The patient was status-post five lumbar spinal surgeries for degenerative disease the most recent of which was an attempted fusion from L1-L5 in January 2015. The patient reported previous infection after surgeries, in the past treated with intravenous (IV) and oral antibiotics without clear information regarding his most recent antibiotic regimen before presenting to our institution.

CT scan of the lumbar spine demonstrated a solid fusion of the L4-5 level. There was evidence of pseudarthrosis at L1-2, L2-3, and L3-4 without solid bony bridging either in the disk spaces or the posterior elements (Figure 1).

**Figure 1:**
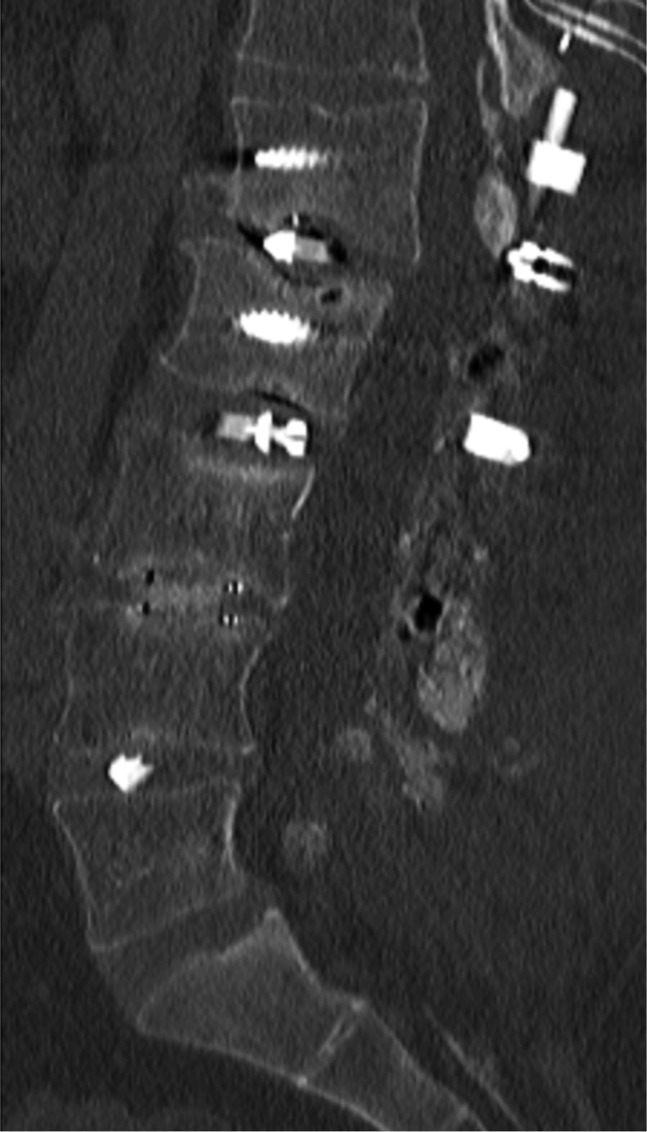
Sagittal CT lumbar spine. Demonstrating no evidence of bony incorporation across the disk space or posterior elements L1-L2 and L2-L3 and incomplete osseous incorporation L3-L4.

The patient was seen in the infectious disease clinic before surgical intervention because of concern of possible on-going infection of his lumbar spine. A preliminary immune and rheumatologic workup was unrevealing. Inflammatory markers including white count, erythrocyte sedimentation rate, and C-reactive protein were all within normal limits. The patient initially underwent implant removal, exploration of the fusion, and irrigation and débridement (I&D) on March 8, 2016. Tissue samples and lumbar spine fluid were obtained. Pseudarthrosis was confirmed at L1-2, L2-3, and L3-4. A residual seroma was evacuated from the tissues deep to the fascia. Owing to the residual fluid collection, the patient was returned to the operating room (OR) on March 15, 2016, for repeat I&D. Repeat tissue and lumbar spine fluid were sent for aerobic, anaerobic, fungal, and acid-fast bacilli (AFB) cultures.

The patient then completed a 6-week course of IV vancomycin for presumed culture negative vertebral osteomyelitis. He continued to have lumbar pain and intermittent fevers during his postoperative course. Magnetic resonance imaging on May 1, 2016, demonstrated a large seroma measuring approximately 13 × 3.6 × 5.6 cm (Figure 2).

**Figure 2:**
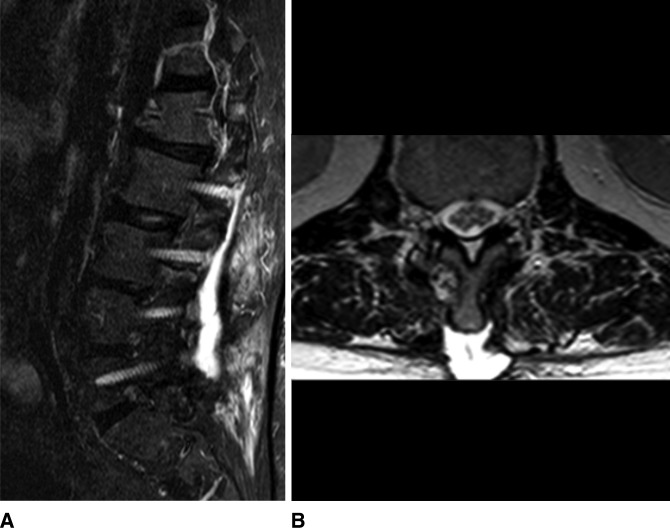
**A**, Sagittal STIR MRI lumbar spine. T2 hyperintense, peripherally enhancing fluid collection extending into pedicle screw tracts. **B**, Axial T2 MRI. Fluid collection in subcutaneous soft tissues extending to T12 spinous process.

Given negative cultures and completion of a six-week course of IV antibiotics, the patient was taken back returned to the OR on March 5, 2016, for T10-pelvis posterior fusion with removal/replacement of the interbody cage at L1-2, T12-L1, and L1-2, Smith-Peterson osteotomies, and a reverse turnover latissimus dorsi muscle flap closure (Figure 3). Repeat surgical cultures were obtained, and Gram stain at that time demonstrated no organisms.

**Figure 3:**
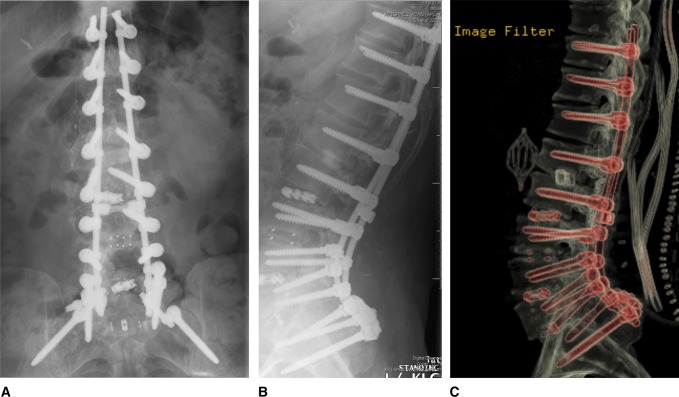
**A** and **B**, AP/lateral lumbar XR. **C**, CT implant series.

During the same hospitalization, the intraoperative deep seroma fluid AFB culture from March 15, 2016, returned positive at eight weeks (May 11, 2016), later identified as *Mycobaterium phlei*. On May 17, 2016, the patient was taken back to the operating room for an I&D, cultures, placement of amikacin calcium phosphate beads, latissimus dorsi flap manipulation, and wound closure.

Histologic analysis of deep tissue surgical specimens collected on May 17, 2016, revealed focal chronic inflammation without granulomas. The patient was started on an empiric 4-drug regimen for nontuberculous mycobacteria (NTM) with intravenous amikacin, oral azithromycin, rifampin, and minocycline postoperatively. Superficial tissue culture isolated from May 17, 2016, during flap débridement did grow rare *Staphylococcus epidermidis*, and the patient was continued on vancomycin during his hospitalization though his oral regimen provided adequate coverage (Figure 4B). His antibiotic treatment was complicated by amikacin-induced ototoxicity and nephrotoxicity along with refractory nausea caused by rifampin. The patient also had severe joint pain when using moxifloxacin as well as levofloxacin. He has since continued on therapy with oral azithromycin and minocycline and elected to maintain on this two drug regimen indefinitely.

**Figure 4:**
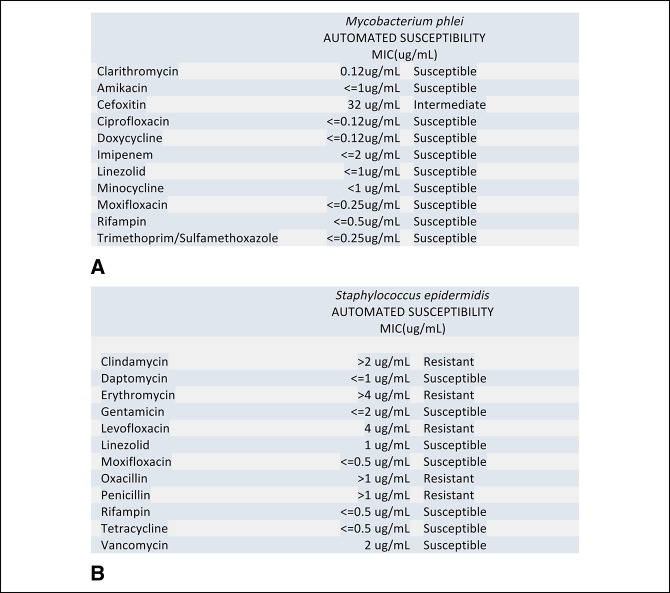
**A**, *Mycobaterium phlei* susceptibility profile—March 5, 2016. **B**, *Staphylococcus epidermidis* susceptibility profile—May 17, 2016.

The patient did have moderate improvement in bilateral radicular symptoms following which is repeated instrumentation and final I&D procedures in May 2016. However, in October 2016, he began to have left-sided radicular symptoms in S1 distribution which was found to be related to the left S1 screw backing out. On October 27, 2016, he was taken to the OR, and the S1 screw was removed. His left-sided symptoms did resolve after this procedure, and bilateral radicular symptoms have improved with addressing the infection, and the most recent CT (December 28, 2018) does demonstrate interval bony bridging (Figure 5).

**Figure 5:**
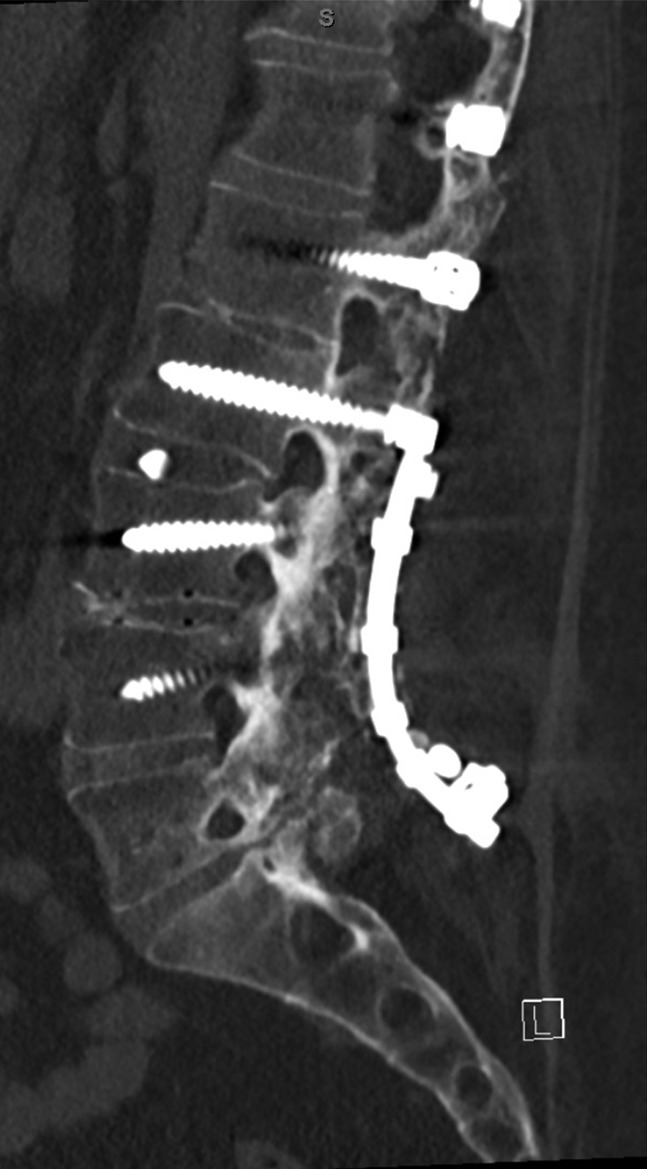
Sagittal CT lumbar spine. Demonstrating bony incorporation across the disk space and posterior elements of lumbosacral spine.

## Discussion

To the best of our knowledge, this is the first reported case description of *M. phlei* osteomyelitis of the spine. *Mycobaterium phlei* is a rapidly growing NTM which is typically nonpathogenic with only four reported cases of human infection.^1,2,3,4^ These include presentations as peritonitis, cardiac implant infection as well as in flexor digitrum, and posterior tibialis tendon of an adult man. The first documented case report of *M. phlei* was found in repeat synovial fluid aspirations of a 7-year-old boy whose symptoms similar to Reiter syndrome, given that he also had associated conjunctivitis and urethritis. Rapidly growing refers to the speed of growth on subculture, which takes approximately 5 days. It grows no more quickly than other mycobacterium, taking 2 to 8 weeks, which was seen in this case on primary recovery media.^5^ Both rapidly growing and slow-growing species of NTM have been implicated in musculoskeletal infections. Most patients have no underlying immune suppression. Diagnosing NTM-related infections is difficult and can often be delayed as conventional microbiologic tests are inadequate. These alone do not allow identification of newly described NTM and may generate inaccurate diagnoses.^6^ Early definitive diagnosis is usually not readily possible because several weeks are required to obtain the results of AFB culture. Though, in the absence of a microbial pathogen, a polymerase chain reaction can be done which can aid in early detection if an adequate amount of specimens are obtained.^7^ Unfortunately, this was not done in our patient, and early detection did not occur. Mycobacterial culture still remains the benchmark for isolation.

Currently, there are no consensus guidelines concerning the treatment of vertebral osteomyelitis caused by NTM because there have been few clinical trials with adequate numbers of patients comparing different therapeutic regimens.^8^ Generally, NTM tend to be resistant to antituberculous drugs and susceptibility vary markedly among species.

Once NTM vertebral osteomyelitis is diagnosed, a combination of surgical débridement and appropriate, individualized antimycobacterial chemotherapy is most effective for treatment.^9^ Surgical débridement and drainage of abscesses are crucial for local NTM osteomyelitis because the mycobacterial burden in the bone marrow is high and the dead bone might provide a storage site for mycobacteria. At the time of making the diagnosis of osteomyelitis, NTM should be included in the differential diagnosis. Although there are no established guidelines for NTM, sufficient time should be allowed for speciation and antibiotic susceptibilities to select the appropriate antimicrobial regimen. NTM osteomyelitis can have a poor prognosis, and physicians should thus be aware of its clinical significance. Since the mechanism of NTM pathogenesis remains poorly understood, there are limited prophylactic options currently.^10^ This case serves as the first description of *M. phlei* osteomyelitis of the spine and as a reminder that, in patients with osteomyelitis caused by undetermined pathogens, care must be taken to differentiate NTM infection from normal pyogenic infection or tuberculosis, through blood tests, imaging studies, pathological examination, AFB tests, and mycobacterial culture as well as specimen biopsies. Doing extensive surgical débridement is key to addressing infection burden and eliminating the offending pathogen. Also, in vitro drug susceptibility testing is important for anti-NTM chemotherapy, and observing adverse reactions is necessary and critical to guiding treatment.
